# Photoinduced Transformations with Diverse Maleimide Scaffolds

**DOI:** 10.3390/molecules29204895

**Published:** 2024-10-16

**Authors:** Jayachandran Parthiban, Dipti Garg, Jayaraman Sivaguru

**Affiliations:** Center for Photochemical Sciences, Department of Chemistry, Bowling Green State University, 141 Overman Hall, Bowling Green, OH 43403, USA; jparthi@bgsu.edu (J.P.); diptig@bgsu.edu (D.G.)

**Keywords:** photocycloaddition, maleimide, selectivity, chemical transformation, methodology, light-mediated, [5 + 2] photocycloaddition, [2 + 2] photocycloaddition

## Abstract

Maleimides serve as crucial components in various synthetic processes and are of significant interest to researchers in bioorganic chemistry and biotechnology. Although thermal reactions involving maleimides have been studied extensively, light-mediated reactions with maleimides remain relatively underutilized. This review focuses on understanding the behavior of maleimides in their excited state, particularly their role as synthetic scaffolds for excited-state reactions. Specific emphasis is placed on the diverse photoreactions involving maleimides and photophysical evaluation from our research group.

## 1. Introduction

**Scheme 1:**
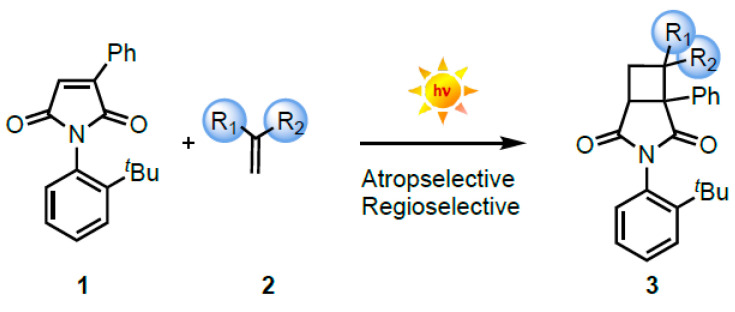
[2 + 2] Photocycloaddition reaction of maleimide with olefin.

Light-driven [2 + 2] cycloaddition reactions continue to attract the attention of chemists with varied scientific interests as they enable easy access to a cyclobutane core [1,2,3]. An attractive feature that adds complexity to these reactions is the generation of potential stereogenic centers [4,5,6]. Historically, [2 + 2] photodimerization reactions involving identical olefins laid the groundwork for [2 + 2] photocycloaddition involving two different alkene partners, leading to a cyclobutane ring [7,8,9,10,11,12,13,14]. Unlike thermal reactions, in photochemical transformations, reaction control in isotropic media is dictated by the short excited-state lifetime(s) of the chromophore. This places significant limitations on controlling the stereochemical outcome during the reaction under ambient conditions [3]. One of the approaches that has been quite successful involves the use of organized media where the confinement of excited chromophores lends a hand in controlling reactivity and selectivity [15,16,17]. The knowledge gained in reaction control by confinement led to the development of various catalytic approaches for asymmetric [2 + 2] photocycloadditions [4,5,6,18,19]. In this regard, our group has utilized the strategy of employing restricted bond rotations in atropisomeric chromophores to control reactivity and selectivity in solution at ambient conditions [20,21]. Recently, we uncovered a new excited-state reactivity of methyl-substituted maleimide that underwent a photo-ene reaction instead of the traditional [2 + 2] photocycloaddition with different alkene partners [22]. This observation motivated us to evaluate the [2 + 2] photocycloaddition of atropisomeric phenyl maleimide **1** with substituted alkenes **2** (Scheme 1) to study the influence of restricted bond rotations on intermolecular transformations. Herein, we disclose a comprehensive overview of the photochemical reactivity developed in our group leading to chemo-, regio-, and stereoselective [2 + 2] photocycloaddition featuring diverse maleimide scaffolds [3,22,23,24,25].

## 2. Light-Mediated Photoreactions of Maleimides

### 2.1. Atrop- and Regioselective [2 + 2] Photoycloaddition

The concerted [2 + 2] cycloaddition reaction between two alkene partners leading to cyclobutane is a thermally forbidden process. To address this limitation, light-initiated [2 + 2] cycloaddition to access cyclobutanes has attracted the attention of various research groups [4,6,18,19,21]. An additional feature of the [2 + 2] photocycloaddition process is that it provides access to four stereogenic centers in the photoproduct. We have demonstrated a viable pathway to access stereo-enriched cyclobutanes via atrop- and regioselective intermolecular [2 + 2] photocycloaddition with atropisomeric phenyl-substituted maleimide (**1**) and olefins (**2**) as coupling partners (Scheme 1). 

**Scheme 2:**
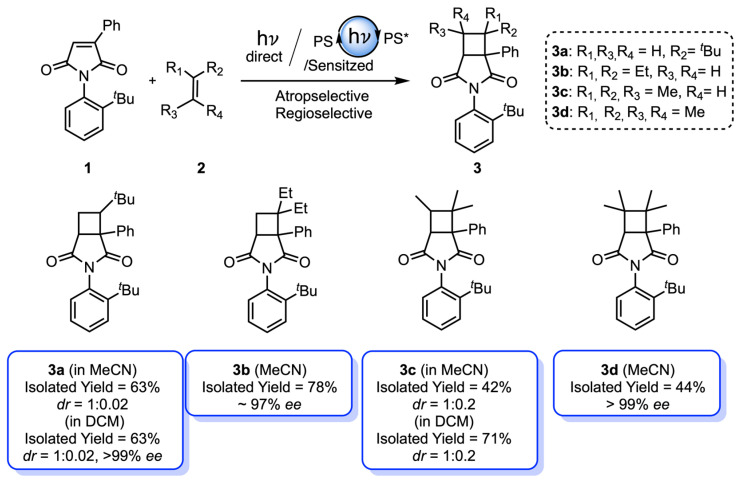
Distribution of photoproducts **3a**–**d** upon photoreaction of atroposelective maleimide **1** with various alkenes **2a**–**d**.

Axial chirality in maleimide **1** arises due to N–C(aryl) bond rotation featuring high free energy for the racemization barrier (ΔG^‡^_rac_ = 29.9 kcal/mol; *k_rac_*@70 °C = 6.1 × 10^−7^ s^−1^) [20]. This high barrier for racemization enabled the successful induction of chirality in the cyclobutane system leading to stereo-enriched photoproduct **3a**–**d** formation (Scheme 2) [3]. The isolated yield in the system varied from 42% to 78% with a diastereomeric ratio varying from 1:0.2 to 1:0.02. For atropoisomeric systems, the enantiomeric excess in the photoproduct was >95%.

**Figure 1:**
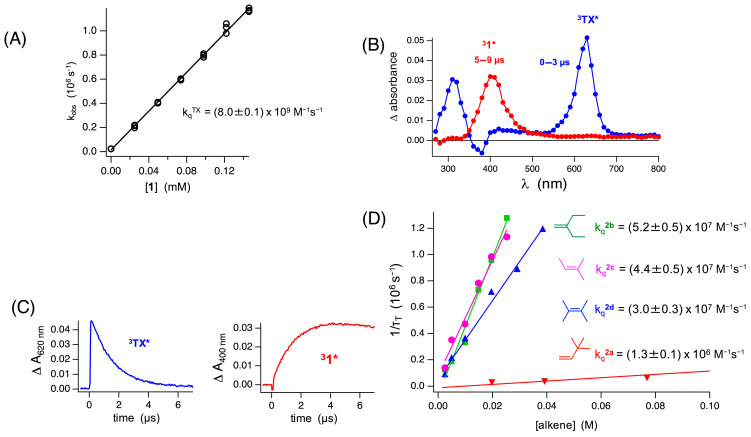
Photophysical studies featuring maleimide **1** with various alkenes **2a**–**d** showcasing an energy transfer mechanism from thioxanthone sensitizer. (**A**) Determination of the quenching rate constant k_q_ of quenching of thioxanthone (Tx) triplet states by **1** using laser flash photolysis. (**B**) Transient absorption spectra after laser flash photolysis of Tx in the presence of **1** (0.1 mM) monitored 0–3 μs (blue) and 5–9 μs (red) after the laser pulse (λ_ex_ = 355 nm, 7 ns pulse width) of argon-saturated MeCN. (**C**) Kinetic traces for LFP experiments showcasing the decay of ^3^Tx^*^ monitored at 620 nm (blue) and formation of ^3^1^*^ monitored at 400 nm (red). (**D**) Determination of the bimolecular quenching rate constant k_q_ for quenching of ^3^1^*^ by various alkenes **2a**–**2d** using laser flash photolysis. Figure adapted from reference [3].

To rationalize the observed reactivity and selectivity in maleimide **1**, mechanistic investigation using laser flash photolysis was performed. As the reaction was performed under sensitized irradiation conditions using thioxanthone (Tx) as the triplet sensitizer, the excited-state quenching dynamics of Tx with maleimides was performed. The triplet-excited ^3^Tx^*^ was quenched by **1** at the near-diffusion control rate that was reflected by the bimolecular quenching rate ($k_{q}^{Tx}$ = 8 ± 0.1 × 10^9^ M^−1^ s^−1^), indicating the feasibility of triplet energy transfer (TEnT) from the excited-state thioxanthone photocatalyst to ground-state maleimide **1** (Figure 1). This conclusion was again supported by plotting the decay kinetics of ^3^Tx^*^ alongside the rising kinetics of ^3^1^*^ that occurred with the same rate. Hence, a plausible mechanism was proposed in which ^3^1^*^ was generated via triplet energy transfer (TEnT). The triplet maleimides reacted with alkenes with bimolecular rate constants that was dependent on the type of alkenes. For mono-substituted alkene **2a,** the bimolecular rate constant for the quenching of ^3^1^*^ was ($k_{q}^{2a}$) 1.3 ± 0.1 × 10^6^ M^−1^ s^−1^, while for the other alkenes, it varied from 3 ± 0.3 × 10^7^ M^−1^ s^−1^ (for tetra-substituted alkene **2d**) to 5 ± 0.5 × 10^7^ M^−1^ s^−1^ (for di-substituted alkene **2b**).

**Scheme 3:**
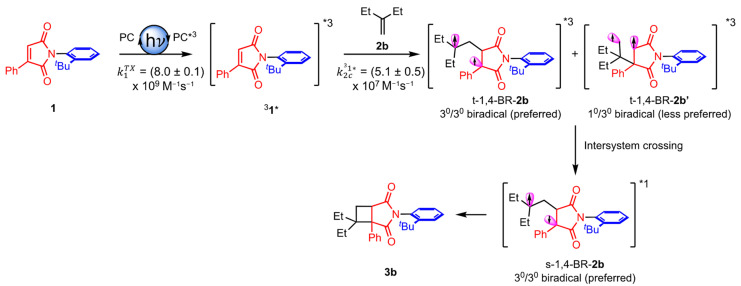
Plausible mechanism of [2 + 2] photocycloaddition reaction between maleimide **1** and alkene **2**.

Based on the photophysical studies (Figure 1), a mechanistic rationale for the observed reactivity and selectivity was proposed featuring triplet-excited-state maleimide ^3^1^*^. This triplet maleimide ^3^1^*^ reacted with alkenes leading to the observed reactivity and selectivity in the [2 + 2] photocycloaddition reaction (Scheme 3). For simplicity, alkene **2b** is depicted as an example which, upon reaction with ^3^1^*^, generates triplet 1,4–biradicals t–1,4BR–**2b** and t–1,4BR–**2b’**, because the stability of t–1,4BR–**2b** was likely preferred (tertiary radical centers) via t–1,4BR–**2b’** featuring secondary radical centers. The triplet t–1,4BR–**2b** diradical intersystem crossed to form the corresponding singlet biradical s–1,4BR–**2b** enroute to the formation of the observed photoproduct **3b**. Thus, the stability of the biradicals controls the formation of the regio-isomeric photoproduct in the system.

### 2.2. Atrop-Selective Intramolecular [2 + 2] Photocycloaddition of Maleimides

**Scheme 4:**
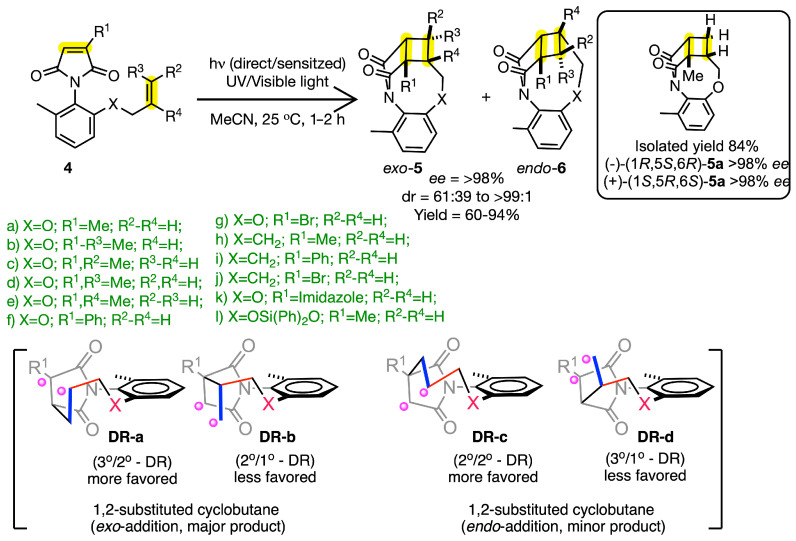
[2 + 2] Photocycloaddition of atropisomeric maleimide. **DR a**–**d** represent the diradical intermediates formed during the reaction. The relative stability of the diradical intermediates influences the product stereochemistry (*endo vs*. *exo*).

As part of transferring chirality from the excited state, our group investigated a study on atroposelective intramolecular [2 + 2] photocycloaddition in maleimide chromophores **4** (Scheme 4). By introducing an alkenyl 4–carbon (butenyl) tether/pendant, it resulted in the exclusive formation of the [2 + 2] photoproduct, demonstrating excellent control over the reactivity. To further advance the atropisomeric design, the strategy was extended to introduce an ether tether (i.e., oxy-allyl chain), which once again resulted in the [2 + 2] photoproduct exclusively. Based on extensive photophysical investigation, this study revealed that the reaction proceeded via a triplet excited state generated either via direct irradiation followed by intersystem crossing or via triplet sensitization generating the triplet maleimide ^3^4^*^. This triplet-excited maleimide led to *exo*–**5** and *endo*–**6** photo-adducts with varying enantiomeric excess (>98% *ee*) for all the substrates studied. Although excellent enantiomeric excess was observed in the photoproduct, the diastereomeric ratio in the product mainly depended on the substitution of the maleimide ring and the alkenyl tether. Based on scrambling experiments of the alkenyl tether on maleimides, they suggested that the external bond in the cyclobutane formed initially, followed by the recombination of the 1,4 biradical, leading to cyclobutane adducts (Scheme 4) [23].

### 2.3. [5 + 2] Photocycloaddition of Maleimides

**Scheme 5:**
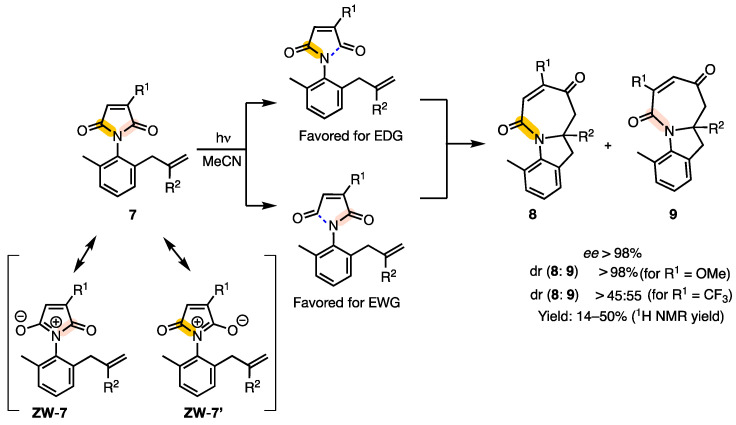
[5 + 2] Photocycloaddition of atropisomeric maleimide derivatives **7.** The N–CO bonds are highlighted in yellow, leading to the formation of **ZW**–**7** and **ZW**–**7′**, which correspond to the **8** and **9** products, respectively.

As part of the investigation of excited-state maleimides, our group also evaluated the possibility of [5 + 2] photocycloaddition. Alkenyl-substituted maleimides **7** underwent [5 + 2] photocycloaddition enroute to the formation of products **8**/**9**. An atroposelective variant of the photoreaction was also developed for the observed [5 + 2] photocycloaddition leading to **8**/**9** with excellent *ee* values (>98%). It was observed that the electron-donating and -withdrawing R^1^ substituent gave the corresponding azepinone-based products **8** and **9**, respectively. The reversal of product selectivity was rationalized based on the stabilization of the zwitterionic forms (**ZW**–**7** and **ZW**–**7′**) of maleimide (Scheme 5) [24].

### 2.4. Photo-ene Reaction of Maleimides

**Scheme 6:**
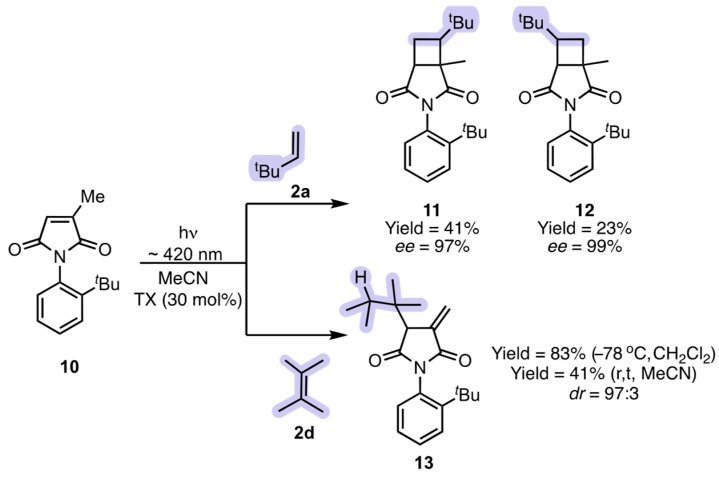
Photo-ene reaction of maleimide derivative **10**.

While [2 + 2] photocycloaddition is a very known process initiated by light, under thermal conditions, alkenes with active allylic hydrogens undergo an ene reaction at elevated temperatures (typically in excess of 130 °C in the absence of any catalyst). Research from our group demonstrated that one can perform a photo-ene reaction by circumventing the known [2 + 2] photocycloaddition process. The photoreaction of maleimide **10** (citraconicimide) with various substituted alkenes resulted in either [2 + 2] photocycloaddition or photo-ene reaction (Scheme 6) [22]. The photoreaction of **10** with various mono- and di-substituted alkenes gave the expected [2 + 2] photocycloaddition products **11** and **12**. On the other hand, the photoreaction of **10** with tri- and tetra-substituted alkenes gave the novel photo-ene product **13** with isolated yields varying from 41% (at room temperature) to 83% (−78 °C). In addition, at low temperature, the diastereomeric ratio was 97:3. Based on detailed photophysical studies, the nature and the lifetime of the intermediates were found to be crucial in determining the photo-ene *vs*. [2 + 2] photocycloaddition pathways. The stability of the intermediates in turn can be fine-tuned by substituents on the alkene reaction partners, offering new venues to explore its potential for diverse photoreactions, such as synergistic-excited-state photocatalysis [22].

### 2.5. Diversity in Photoreactions of Maleimides—An Overview

**Scheme 7:**
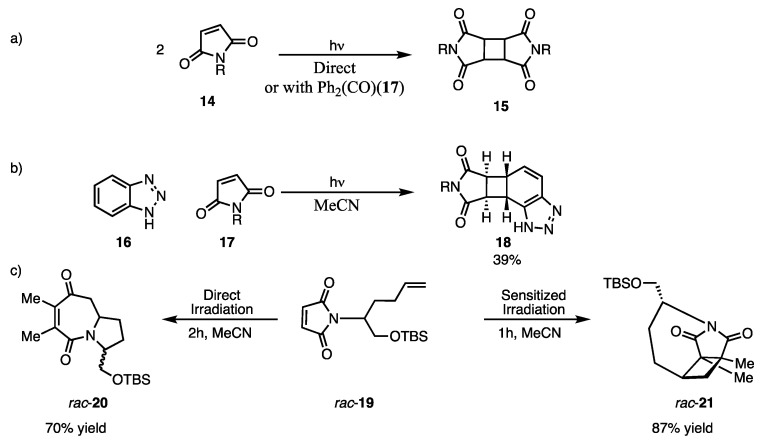
Earlier reports on maleimide photocycloaddition. (**a**) Dimerization of maleimide **14**. (**b**) Intermolecular photocycloaddition of maleimide **17**. (**c**) Photocycloaddition of maleimide *rac*-**20**.

Since the early 1960s, multiple research groups have investigated the photodimerization of maleimide and published some of the earliest findings on maleimide photochemistry. In 1982, Bong et al. conducted a study on the photodimerization of maleimide. They found that subjecting maleimide to UV light (~350 nm) for 10 h, either directly or through sensitized irradiation using benzophenone or acetophenone as a sensitizer, resulted in efficient photoreaction (Scheme 7a) [26]. In 2001 and 2008, Booker–Milburn employed maleimides for [2 + 2] and [5 + 2] photocycloadditions to synthesize intricate organic molecules with stereogenic centers such as perhydroazaazulene or stamina alkaloids. This research illustrates that *N*–alkenyl maleimides demonstrate varying reactivity in sensitized and non-sensitized reactions due to the participation of different bonds, such as C–N and C–C bonds within the same molecule. This led to the formation of different products, including 7,5-fused azepines **20** or cyclobutanes **21**, through two distinct modes of photocycloaddition: [5 + 2] photocycloaddition [27] and [2 + 2] photocycloaddition [28] (Scheme 7b,c).

**Scheme 8:**
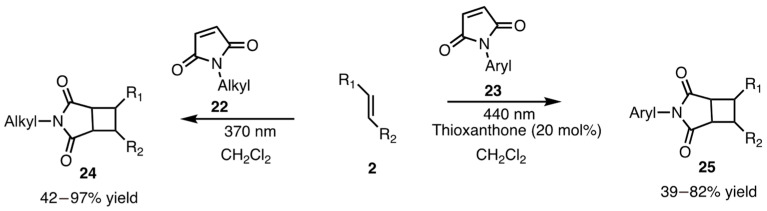
Maleimide alkyl vs. aryl reactivity on photocycloaddition.

In 2023, Kokotos and coworkers [29] investigated the diverse reactivity of maleimide by employing a sensitizer and manipulating the excited state to control the outcome of the product selectivity. [2 + 2] Photocycloadditions of *N*–alkyl maleimides **22** reacted with alkenes when directly exposed to 370 nm UV light, whereas *N*–aryl maleimides **23** required a sensitizer (Tx) and 440 nm visible light for the reaction (Scheme 8). Quantum yield measurements revealed that *N*–aryl maleimides had a zero-triplet quantum yield, necessitating a photosensitizer to transfer triplet energy to the substrate for product formation. On the other hand, the triplet quantum yield of *N*–alkyl maleimides improved in the presence of TFA or HFIP, enabling product formation through direct irradiation. This showed the uniqueness of the maleimide scaffold in dictating photochemical reactivity. 

There is extensive research on the use of maleimides as photoinitiators for photopolymerization due to their unique properties in generating radicals and initiating polymerization. Decker et al. [30] investigated the photopolymerization of a diacrylate monomer by employing maleimides (*N*–aliphatic maleimide) as photoinitiators to initiate chain-growth polymerization. They showed that the maleimides unlike common photoinitiators could act both as a polymerizable monomer and as an initiator, particularly with vinyl ethers, acrylates, and styrene oxides. Their preliminary studies suggested that excited-state maleimides were effective as H-abstracting agents, generating radical species that was responsible for initiating polymerization. This unique dual function of maleimides has increased interest in maleimides within the UV curing field.

## 3. Conclusions

Maleimides are versatile scaffolds that have been widely employed in synthetic and materials chemistry. Research from our group and others have demonstrated the rich excited-state chemistry of maleimides that can be exploited for various applications. As maleimides have been widely used in pharmaceutical and materials applications and are also well-established building blocks in synthetic organic chemistry, their excited-state chemistry will be of contemporary interest to chemists across disciplines [31,32]. We have also discussed various maleimide-based reactions from other research groups, highlighting their diverse reactivity. Furthermore, we included the role of maleimides as photoinitiators for photopolymerization, using specific examples to illustrate their utility in initiating polymerization reactions. The prevailing gap in the excited-state chemistry of maleimides coupled with their diverse applications are fertile grounds for exploration in basic and applied research.
